# Maternal and Gestational Risk Factors for Hypospadias

**DOI:** 10.1289/ehp.10791

**Published:** 2008-04-09

**Authors:** Olof Akre, Heather A. Boyd, Martin Ahlgren, Kerstin Wilbrand, Tine Westergaard, Henrik Hjalgrim, Agneta Nordenskjöld, Anders Ekbom, Mads Melbye

**Affiliations:** 1 Clinical Epidemiology Unit, Department of Medicine, Karolinska University Hospital, Stockholm, Sweden; 2 Department of Epidemiology Research, Statens Serum Institut, Copenhagen, Denmark; 3 Department of Public Health and Caring Sciences, Uppsala University, Uppsala, Sweden; 4 Department of Molecular Medicine and Surgery and; 5 Division of Pediatric Surgery, Department of Women and Child Health, Karolinska Institutet, Stockholm, Sweden

**Keywords:** birth weight, body mass index, diet, hypertension, hypospadias, pregnancy, risk

## Abstract

**Background:**

An increase in the prevalence of hypospadias has been reported, but the environmental causes remain virtually unknown.

**Objectives:**

Our goal was to assess the association between risk of hypospadias and indicators of placental function and endogenous hormone levels, exposure to exogenous hormones, maternal diet during pregnancy, and other environmental factors.

**Methods:**

We conducted a case–control study in Sweden and Denmark from 2000 through 2005 using self-administered questionnaires completed by mothers of hypospadias cases and matched controls. The response rate was 88% and 81% among mothers of cases and controls, respectively. The analyses included 292 cases and 427 controls.

**Results:**

A diet during pregnancy lacking both fish and meat was associated with a more than 4-fold increased risk of hypospadias [odds ratio (OR) = 4.6; 95% confidence interval (CI), 1.6–13.3]. Boys born to obese [body mass index (BMI) ≥ 30] women had a more than 2-fold increased risk of hypospadias (OR = 2.6; 95% CI, 1.2–5.7) compared with boys born to mothers with a normal weight (BMI = 20–24). Maternal hypertension during pregnancy and absence of maternal nausea increased a boy’s risk of hypospadias 2.0-fold (95% CI, 1.1–3.7) and 1.8-fold (95% CI, 1.2–2.8), respectively. Nausea in late pregnancy also appeared to be positively associated with hypospadias risk (OR = 7.6; 95% CI, 1.1–53).

**Conclusions:**

A pregnancy diet lacking meat and fish appears to increase the risk of hypospadias in the offspring. Other risk associations were compatible with a role for placental insufficiency in the etiology of hypospadias.

Hypospadias is a male birth defect in which the urethral orifice is abnormally located on the ventral side of the penis. In Western countries, the prevalence of hypospadias varies from 2 to 8 cases per 1,000 live births, with increases in prevalence reported over time in several populations (Czeizel et al. 1986; Kallen and Winberg 1982; Nelson et al. 2005; Paulozzi 1999; Paulozzi et al. 1997; Pierik et al. 2002). Although we know that urethral development, occurring during gestational weeks 7–16, is regulated by androgens, the causes of most hypospadias cases are unknown (Baskin and Ebbers 2006). Hypospadias is only rarely a monogenic trait (Fredell et al. 2002). Several gene defects, including defects in genes coding for the androgen receptor, are associated with hypospadias but account for only a small proportion of cases (Nelson et al. 2005).

Reported associations between hypospadias and low birth weight, preterm birth, and signs of preeclampsia (Akre et al. 1999; Aschim et al. 2004; Boisen et al. 2005; Hussain et al. 2002; Main et al. 2006) indicate that placental malfunction and subsequent abnormalities in hormone regulation and/or the provision of nutrients to the fetus may play a role in the maldevelopment of the urethra. Increases in prevalence, as reported by several investigators (Paulozzi 1999; Paulozzi et al. 1997; Pierik et al. 2002; Toppari et al. 2001), also point to the involvement of environmental determinants. To date, only a few studies have reported positive associations between hypospadias and environmental exposures, but reports of links with prenatal exposure to diethylstilbestrol (Brouwers et al. 2006; Klip et al. 2002) and exogenous progestins (Carmichael et al. 2005a), as well as of increased risk associated with maternal vegetarian diet (North and Golding 2000), are of interest. With the results of these previous studies in mind, in the present case–control study we investigated the relationships between hypospadias risk and maternal diet, exposure to exogenous hormones, and exposures thought to reflect placental function.

## Methods

The data for this study were collected as part of a joint Danish–Swedish case–control study of both hypospadias and cryptorchidism. Virtually identical questionnaires were used in the two countries to collect exposure information from the mothers of cases and controls. However, case ascertainment and control selection differed in Denmark and Sweden; consequently, we describe study subject recruitment separately for each country.

The study complied with all applicable international regulations and was approved by ethical review boards in both Sweden and Denmark, and the subjects gave informed consent before the study.

### Recruitment of hypospadias cases and controls: Sweden

Each Swedish administrative region has a single pediatric surgery clinic to which all hypospadias cases are referred upon discovery. We recruited hypospadias cases without concomitant malformations or chromosomal abnormalities from the clinics at Karolinska University Hospital (Stockholm) and Uppsala Academic Hospital, which together serve a population with almost 50,000 live births per year. We invited mothers of boys with a hypospadias diagnosis, confirmed by a pediatric urologist in the periods March 2000 through September 2001 and September 2003 through March 2005, to participate in our case–control study; 204 (95%) of 214 women accepted the invitation. These 214 cases correspond to an approximate prevalence at birth of 0.0015. Although the estimated prevalence at birth of hypospadias in Sweden is 0.0033, our case detection rate seems reasonable, because we restricted the study to isolated malformations that were discovered at an early age. Participating mothers completed a self-administered questionnaire (see below) during the clinic visit at which they were recruited into the study. A study coordinator was available at that time to answer any questions the mothers had about the questionnaire but was otherwise uninvolved in its administration.

For each hypospadias case, we randomly selected one or two control boys without known malformations or chromosomal abnormalities from the Swedish Medical Birth Register (one control per case during the first half of the study, and two controls per case during the second half), born within 6 months of and in the same county as the case. We obtained their addresses from the Swedish Tax Authority’s Population Register (Statistics Sweden, Örebro, Sweden; http://www.scb.se) and mailed an information packet and a questionnaire to each control’s mother. Shortly thereafter, we contacted the women by phone to encourage participation and answer any questions. Of 421 approached control mothers, 353 (84%) returned a completed questionnaire.

In Sweden, the median age of the son when his mother completed the questionnaire was 2 months (range, 0–6 months) for cases and 5 months (range, 1–9 months) for controls.

### Recruitment of hypospadias cases and controls: Denmark

#### Subjects from the Danish National Birth Cohort

The Danish National Birth Cohort (DNBC) is a population-based cohort of Danish women and children established to allow investigation of the impact of a wide range of *in utero* exposures on health over the life course (Olsen et al. 2001). More than 101,000 pregnant women recruited during 1996–2002 gave birth to nearly 94,000 children between 1997 and 2003. The women were interviewed twice while pregnant and twice after delivery. For boys, the first postpartum interview, which occurred when the infant was approximately 6 months of age, included questions about hypospadias as well as other birth defects and chromosomal abnormalities.

Within 1 month of the interview, we mailed study materials to DNBC mothers who had their first postpartum interview between December 2000 and February 2003 and responded that their son had been diagnosed with hypospadias, but not with Down syndrome, cleft lip and/or palate, or cryptorchidism. We then contacted them by phone to ask them to participate in our study. We confirmed the diagnosis of hypospadias by the mother’s response to the following specific question in our exposure-measurement questionnaire: Has a doctor stated that your son’s urethral orifice is aberrantly located on the underside of his penis (the condition is also called hypospadias)?

For each case, we randomly selected two controls boys from DNBC cohort boys meeting the following criteria: *a*) never diagnosed with hypospadias, cryptorchidism, Down syndrome, or cleft lip/palate (according to information provided by their mothers in the first DNBC postpartum interview); *b*) born in the same county as the case; and *c*) born within 2 weeks (maximum) of the case. As with case mothers, we mailed study materials to control mothers within 1 month of the DNBC interview and then contacted them by phone.

#### Subjects from the Danish national registers

To increase the power of our study, we recruited additional cases and controls through the National Patient Register and the Medical Birth Register (National Board of Health, Copenhagen; http://www.sst.dk). The National Patient Register contains all discharge diagnoses for inpatients discharged from Danish hospitals, as well as all diagnoses associated with hospital outpatient visits. The Medical Birth Register contains information on all births occurring in Denmark. Both registers contain mandatorily reportable information, are updated daily, and are considered close to complete. We obtained information on newly registered cases of hypospadias [*International Classification of Diseases*, *10th Revision* (ICD-10), codes Q54.0–Q54.9 (World Health Organization 1993)] in boys < 6 months of age from the National Patient Register every 3 months between December 2001 and February 2003. We mailed study materials to mothers of cases without concomitant Down syndrome, cleft lip and/or palate, or cryptorchidism, and then contacted them by phone and asked them to participate in our study.

For each case, we selected one control boy at random from the boys listed in the Medical Birth Register as being born in the same county and calendar month as the case. We used information from the National Patient Register to confirm that controls had never been diagnosed with hypospadias, cryptorchidism, Down syndrome, or cleft lip and/or palate. We contacted mothers of control boys in the same way as case mothers.

In total, 138 (80%) of 173 case mothers we contacted in Denmark and 182 (75%) of 243 invited controls returned a completed questionnaire. Forty-one (30%) of the cases and 52 (29%) of the controls in Denmark came from the DNBC. The median age in Denmark of the son when his mother completed the questionnaire was 5 months (range, 1–8 months) for cases and 6 months (range, 2–7 months) for controls.

### Collection of exposure data

Each mother completed a self-administered questionnaire that included questions about her gynecologic and reproductive history, her health and diet before and during the index pregnancy, and her smoking habits while pregnant, as well as parental demographic, physical, and social characteristics and characteristics of the case/control boy. We received completed questionnaires for 342 cases and 535 controls. Apart from a few extra questions in the Danish questionnaire regarding the ascertainment of hypospadias, the questionnaires used in Sweden and Denmark were identical.

For most questions, the questionnaire provided two or more choices of response. Some of the less intuitive questions are described here. We assessed irregular menstrual periods by asking whether the mother always had regular periods (yes/no). In the question about menstrual cycle length, we gave different intervals; possible responses also included unknown cycle length and irregular periods. We assessed contraceptive use at conception by listing seven types of contraception: combined oral contraceptives, minipills, injections, implants, intrauterine device (IUD), IUD with hormones, and other methods such as condom and natural methods. We ascertained history of complications such as high blood pressure and proteinuria with yes/no questions. In the question regarding nausea during pregnancy, we asked those who stated that they had been nauseous to specify the trimester of nausea, and permitted them to specify more than one trimester. We assessed meat consumption by asking whether meat had been consumed at least once a week, whereas the consumption of fish we further categorized into less than once weekly, once or twice, three or four times, and more than four times weekly. Because of a paucity of mothers in the highest category, however, we collapsed the top two categories.

### Statistical analyses

#### Exclusions

In our questionnaire, we asked mothers of boys recruited from the DNBC to confirm that their sons had (cases) or did not have (controls) hypospadias. We also asked them to confirm that their sons did not have cryptorchidism. We excluded cases from the analysis if *a*) their mothers did not answer the hypospadias question (*n* = 10) or denied that their sons had hypospadias (*n* = 3), and no hypospadias diagnosis code was registered in the National Patient Register; *b*) information on cryptorchidism status was missing (*n* = 3); or *c*) they were reported to have cryptorchidism (*n* = 5). We excluded controls if *a*) information on hypospadias (*n* = 4) or cryptorchidism (*n* = 5) was missing, *b*) their mothers claimed they had hypospadias (*n* = 1), or *c*) they were reported to have cryptorchidism (*n* = 4). After exclusion, 321 cases and 522 controls remained for analysis.

#### Modeling strategy

We performed analyses using Intercooled Stata 8.1 for Microsoft Windows (StataCorp., College Station, TX, USA) and SAS software (version 9.1; SAS Institute Inc., Cary, NC, USA). We estimated odds ratios (ORs) and 95% confidence intervals (CIs) using conditional logistic regression, taking into account the matched design; 291 strata (291 cases, 427 controls) had complete information and were included in the analyses. Variables that were associated with the outcome in univariate analyses or that we regarded as potential confounders (e.g., maternal age, weight for gestational age, gestational age) we considered in multivariable models. Variables that were not associated with the outcome in multivariable analyses and whose withdrawal from the model did not change the OR estimate for at least one other variable in the model by ≥ 10% we did not retain in the final multivariable model. Variables that did not meet the criteria for inclusion in the multivariable model but were of particular interest *a priori* (e.g., contraceptive use at conception, maternal smoking in the first trimester) we added to the final model individually for evaluation in the multivariable setting.

Swedish hypospadias cases recruited by the Stockholm clinic (*n* = 156) were categorized according to severity by a single pediatric urologist (A.N.). To investigate the effects of our exposure variables in the most severely affected group of hypospadias cases possible, we repeated the analyses restricted to more severe cases from this clinic (*n* = 77) and their matched controls. We defined “severe” hypospadias in this case as having the urethral orifice proximal to the glandular corona.

## Results

Table 1 presents variable distributions for cases and controls, along with unadjusted ORs and 95% CIs, for selected study variables. In the univariate analyses, we found the following variables to be positively associated with risk of hypospadias: low maternal age, high maternal body mass index (BMI), low maternal education, pregnancy hypertension, proteinuria during pregnancy, lack of nausea during pregnancy, no meat consumption during pregnancy, no or low intake of fish during pregnancy, maternal passive smoking during pregnancy, low birth weight, low gestational age at birth, and neonatal jaundice (Table 1). Use of nonhormonal forms of contraception at the time of conception was negatively associated with hypospadias risk (Table 1).

In the multivariable analyses, the association with nonhormonal contraceptive use remained, although with wider CIs that included the null (Table 2). Likewise, maternal age, maternal education, and proteinuria were not significantly associated with hypospadias risk in the final model. On the other hand, the association with late-pregnancy nausea became stronger and significant after multiple adjustment. The association between low consumption of meat and risk of hypospadias was also stronger after adjustment.

### Maternal characteristics

Boys born to overweight (prepregnancy BMI = 25–29) or obese (prepregnancy BMI ≥ 30) women were 73% and 162% more likely, respectively, to have hypospadias than were boys born to women with prepregnancy BMIs in the normal range (BMI = 20–24) (adjusted ORs with 95% CIs in Table 2); as maternal prepregnancy BMI increased, the risk of hypospadias also increased (*p*-value for trend = 0.0079).

### Characteristics of the index pregnancy

Maternal hypertension during pregnancy doubled a boy’s risk of hypospadias (Table 2). Compared with maternal nausea in the first trimester, the absence of maternal nausea during pregnancy almost doubled the risk of hypospadias, whereas boys whose mothers experienced nausea only in the third trimester had a significant 7-fold increased risk (Table 2). Passive maternal exposure to tobacco smoke during pregnancy increased the risk of hypospadias by 88%. In contrast, active maternal smoking in the first trimester was not significantly associated with hypospadias risk.

Compared with a maternal diet that included meat and/or fish, a diet lacking both protein sources was associated with a more than 4-fold increased risk of hypospadias (Table 2). When we examined meat and fish consumption separately, boys whose mothers ate meat at least once a week while pregnant were less likely to have hypospadias than were boys whose mothers never ate meat (OR = 0.42; 95% CI, 0.21–0.87). Compared with boys whose mothers ate fish once or twice a week, those whose mothers never (OR = 1.4; 95% CI, 0.84–2.2) or rarely (OR = 2.7; 95% CI, 1.3–5.5) ate fish were more likely to have hypospadias.

### Characteristics of the child at birth

Being small for gestational age (weight for gestational age < 10th percentile) was strongly associated with hypospadias risk, with the smallest boys (< 2.5th percentile) being 4.4 (95% CI, 1.2–15) times as likely to have hypospadias as boys in the 50th to 74th weight for gestational age percentiles (Table 2). Being born preterm (≤ 36 gestational weeks) was associated with a 9-fold increased risk of hypospadias, compared with being born in gestational week 40 or 41 (Table 2).

### Subgroup analyses

When we restricted our analysis by excluding cases with cleaved prepuce or glandular or coronary hypospadias from the Stockholm clinic and their matched controls, we obtained results similar to those presented in Table 2, albeit with wider CIs (data not shown).

## Discussion

This study, one of the largest of its kind, had several important findings. A pregnancy diet lacking in meat and fish, a high maternal prepregnancy BMI, and absence of nausea during early pregnancy all independently increased hypospadias risk.

Our maternal diet finding complements a previously reported strong positive association between maternal vegetarian diet and hypospadias risk (North and Golding 2000). It has previously been suggested that the risk of genital anomalies in male offspring may be increased by intake of different soy proteins frequently ingested by vegetarians. Soybeans contain phytoestrogens that may produce estrogenic as well as antiestrogenic effects via the estrogen receptor (Rosselli et al. 2000). It has been suggested that phytoestrogens from soybeans, for instance, disrupt the masculinization of the male through interference with the pituitary–gonadal axis (Sharpe and Skakkebaek 1993). Alternatively, however, the exclusion of animal proteins could increase the risk of a transient deficiency of some nutrient essential during organogenesis or placentation.

Several authors have suggested that hypospadias may be caused by abnormal levels of pregnancy hormones resulting from impaired placental function or exposure to disruptive exogenous hormones (Akre et al. 1999; Czeizel et al. 1979; Klip et al. 2002). In particular, reduced levels of human chorionic gonadotropin (hCG) have been suggested as a candidate in the etiology of hypospadias (Czeizel et al. 1979). hCG, which is produced by the placenta and is chemically similar to luteinizing hormone, is important to sexual differentiation in the fetus and stimulates the fetal testis before the fetus’s own pituitary–gonadal axis is established. hCG has also been shown to be an angiogenic factor during early pregnancy (Zygmunt et al. 2002). As discussed in more detail below, our findings of associations between an increased risk of hypospadias and no maternal nausea during early pregnancy, low weight for gestational age, preterm birth, gestational hypertension, and maternal overweight may all potentially be explained by impaired placental hormone production.

Nausea in early pregnancy is believed to be caused by the early surge of pregnancy hormones, particularly hCG; the onset and peak of nausea and vomiting in early pregnancy parallel the hCG curve (Furneaux et al. 2001; Verberg et al. 2005). First-trimester nausea is considered to be a sign of a healthy pregnancy, because it is associated with lower risks of miscarriage and premature birth (Cnattingius et al. 2000; Furneaux et al. 2001). In contrast, the absence of nausea may reflect potential problems with the pregnancy and is associated with low hCG levels (Cnattingius et al. 2000; Verberg et al. 2005). Our finding that the absence of nausea during early pregnancy was associated with increased hypospadias risk therefore lends support to the hypothesis that abnormally low hCG levels resulting from placental insufficiency in early pregnancy may be involved in the etiology of hypospadias. Carmichael et al. (2007) reported a similar negative (but nonsignificant) association between early nausea and hypospadias. Our observation that nausea only during late pregnancy (“abnormal” nausea) was associated with an increased risk of hypospadias is more difficult to explain, because the closing of the urethral orifice occurs early in pregnancy. The finding was, however, based on few subjects and may be spurious. On the other hand, little is known about the mechanisms underlying nausea in late pregnancy, but its etiology is likely to differ from that of nausea in early pregnancy, and it may reflect some process initiated at the beginning of pregnancy that has some bearing on the development of hypospadias.

Several previous studies have found associations between hypospadias risk and preterm birth and low birth weight (Akre et al. 1999; Aschim et al. 2004; Moller and Weidner 1999). Many authors have suggested that disturbance of placental function early in pregnancy is a key mechanism underlying both preterm birth/low birth weight and the improper closure of the urethra, because the placenta is the main producer of pregnancy hormones in early pregnancy and is thus instrumental in the differentiation and development of the fetal organs (Akre et al. 1999; Aschim et al. 2004; Boisen et al. 2005; Hussain et al. 2002; Kallen 1988; Weidner et al. 1999). We, too, found associations between hypospadias risk and preterm birth (< 37 weeks gestation) and/or being small for gestational age (< 10th percentile). Our additional finding that hypospadias risk is associated with gestational hypertension, which in turn is associated with preeclampsia and placental dysfunction (Ros et al. 1998), has also previously been noted (Aschim et al. 2004; Sorensen et al. 2005) and provides further support for the notion that placental dysfunction is involved in the etiology of hypospadias.

In the same vein, maternal overweight is known to be associated with increased risks of preeclampsia and late fetal death, possibly as a consequence of vasoconstriction in the placenta (Cnattingius et al. 1998; Eskenazi et al. 1991). In a recent study, Stewart et al. (2006) reported that obese women have significantly lower plasma levels of plasminogen activator inhibitor-2 (PAI-2) during the first trimester of pregnancy, but not thereafter. PAI-2, which is part of the plasminogen activator system, is derived from the functioning placenta and thus a marker of placental function. The authors concluded that their finding may provide a mechanistic link between obesity and preeclampsia. The plasminogen activator system is believed to be important in angiogenesis, and it has also been suggested to be involved in the pathogenesis of congenital malformations. In our data, we found an increased risk of hypospadias among the offspring of overweight and obese women, with a dose–response relationship between BMI and hypospadias risk. It seems plausible that our findings of increased risks of hypospadias associated with both gestational hypertension and obesity, despite their statistical independence, share pathogenetic mechanisms, possibly through their link to impairment of placental function and growth during early pregnancy. Our finding of increased risk associated with maternal overweight is in accordance with recent data from Carmichael et al. (2007).

Previous studies have yielded conflicting results regarding parental smoking (preconception and/or during pregnancy) and the risk of hypospadias (Akre et al. 1999; Brouwers et al. 2007; Carmichael et al. 2005b; Pierik et al. 2004). We found an association between hypospadias risk and exposure to passive smoking, but no association with active maternal smoking. Although there are mechanisms to explain an association with paternal smoking in the absence of an association with maternal smoking—for example, smoking may cause germline mutations—the most likely explanation for the association with paternal smoking is perhaps chance variability. The lack of association with active smoking could, however, be partly explained by nondifferential under-reporting driving the results toward the null.

Unfortunately, we lacked power to study the potential role of hormonal contraception at the time of conception. Those using non-hormonal contraceptive methods such as condoms and “natural” methods had a significantly decreased OR for the risk hypospadias in the crude analysis, but not in the multivariable analysis. Nonhormonal contraceptive methods per se are unlikely to be protective against hypospadias, but women employing them should have less exposure to exogenous hormones in the period just before conception than those who become pregnant immediately after stopping oral contraceptive use or while still using oral contraceptives.

Differences in case and control ascertainment arose because of infrastructure differences between the countries and may raise concerns about the validity of our study. However, although different, study subject ascertainment in each country was population based and should have yielded nearly complete ascertainment of cases in the relevant geographic areas during the time periods in question, along with a representative sample of controls from those areas.

Case ascertainment in Sweden involved examination by a pediatric urologist, thus ensuring the specificity of the hypospadias diagnosis among the Swedish cases. Two-thirds of the cases in Denmark we identified through nationwide registers, using ICD codes for hypospadias. Because reimbursement of the hospital depends on the reporting of these diagnostic codes, little underreporting occurs, although, of course, only detected cases will be reported. Although the sensitivity of the registers for the milder forms of hypospadias may be low, registration of the more severe hypospadias is probably fairly complete. Furthermore, it is unlikely that boys are falsely registered with the condition (high specificity of the registers for hypospadias). The remaining third of the cases from Denmark we identified based on information from the mother herself. We find it unlikely that a woman would have mistakenly stated that her son had the condition. (There may, however, have been underreporting of milder forms of the condition.) Therefore, we believe that the specificity of our case ascertainment methods for the diagnosis of hypospadias was high in Denmark as well as in Sweden.

The sensitivity of our case ascertainment methods is less important for the internal validity of the study than for the generalizability of the results. Our case group may have been skewed toward more severe cases because of the requirement for confirmation of the diagnosis by a pediatric urologist in Sweden, the presumed tendency for registered cases in Denmark to be more severe, and the fact that we limited our case group to very young boys (thereby possibly missing cases detected at later ages). Because we enrolled Swedish cases at a lower average age than the Danish cases, the Swedish cases in particular may be of somewhat higher grade. However, when we restricted the analysis to Swedish cases of known severity and focused on more severe forms of hypospadias, we found no indication of different etiologic patterns for the severe cases that could indicate that our results are not generalizable to less severe cases, nor did we see any clear sign of a dose–response relation between any exposure and degree of hypospadias.

The response rate for Swedish case mothers was exceptionally high (95%), because they were approached in person in the clinics. However, the response rates for Swedish control mothers and Danish case and control mothers, whom we recruited by telephone, were also very good (75–84%). Because the response rates in all four groups were high, any bias due to nonparticipation is likely to be negligible, and the chance of selection bias due to differences in recruitment methods is minimal.

The use of self-administered questionnaires may have introduced some misclassification of exposure; however, any such misclassification should not differ between cases and controls, and any bias thus introduced should be in the direction of the null. Bias due to differential recall of exposure between cases and controls is a well-known phenomenon in malformation studies. However, because there are no well-established etiologies for hypospadias, for most exposures mothers of cases would have been unlikely to systematically over- or underreport their exposure status compared with control mothers. In fact, the directions of some of our associations are the opposite of what might have been expected if recall bias had influenced the results. For example, it is unlikely that lack of nausea is perceived by laymen to be a problem, making it improbable that our finding of an increased risk of hypospadias associated with lack of nausea could be explained by differential recall. Similarly, a vegetarian diet is often considered part of a healthy lifestyle, and associations between adverse outcomes and a vegetarian diet are thus not likely to be a result of recall bias.

In conclusion, consistent with a previous hypothesis-generating study (North and Golding 2000), we found a strongly increased risk of hypospadias associated with a maternal diet lacking in fish and meat. Furthermore, we found positive associations between the absence of nausea during pregnancy, maternal hypertension, high BMI, and risk of hypospadias. Most of these findings are compatible with a role for placental insufficiency in the etiology of hypospadias.

## Figures and Tables

**Table 1:** Associations between hypospadias and selected maternal, pregnancy, and birth characteristics: unadjusted ORs from a matched case–control study, Denmark and Sweden, 2000–2005.

	Cases (*n* = 292)	Controls (*n* = 427)			Cases (*n* = 292)	Controls (*n* = 427)	
Variable	No. (%)	No. (%)	OR (95% CI)	Variable	No. (%)	No. (%)	OR (95% CI)
Maternal characteristics				Proteinuria			
Age (years)				No	252 (87)	399 (93)	1
18–24	30 (10)	27 (6.3)	1.6 (0.88–2.8)	Yes	39 (13)	28 (6.6)	2.3 (1.4–3.9)
25–29	89 (31)	110 (26)	1.1 (0.78–1.7)	Nausea			
30–34	114 (39)	167 (39)	1	No	131 (45)	141 (33)	1.9 (1.3–2.6)
≥ 35	58 (20)	123 (29)	0.66 (0.44–1.00)	Early pregnancy only^d^	132 (46)	254 (59)	1
Height [cm (quartiles)]				Late pregnancy only^3^	6 (2.1)	4 (0.9)	3.9 (0.94–17)
≤ 163	78 (27)	97 (23)	1	Early and late pregnancy	20 (6.9)	28 (6.6)	1.3 (0.70–2.6)
164–167	65 (22)	103 (24)	0.77 (0.49–1.2)	Fever, months 1–3			
168–171	72 (25)	111 (26)	0.80 (0.52–1.2)	No	267 (91)	386 (91)	1
≥ 172	76 (26)	115 (27)	0.82 (0.54–1.2)	Yes	25 (8.6)	39 (9.2)	0.90 (0.53–1.5)
BMI, prepregnancy (kg/m^2^)				Weekly meat consumption			
≤ 19	49 (17)	71 (17)	1.3 (0.83–1.9)	No	29 (10)	28 (6.6)	1.6 (0.9–2.7)
20–24	151 (52)	257 (61)	1	Yes	263 (90)	399 (93)	1
25–29	58 (20)	70 (17)	1.6 (1.05–2.5)	Weekly fish consumption			
≥ 30	32 (11)	22 (5)	2.7 (1.5–4.9)	None	66 (23)	78 (18)	1.7 (1.1–2.5)
Education (years)				Less than once	70 (24)	60 (14)	2.5 (1.4–4.5)
≤ 9	42 (14)	46 (11)	1.5 (0.93–2.4)	Once or twice	149 (51)	269 (63)	1
10–12	120 (41)	155 (37)	1.2 (0.88–1.8)	More than twice	6 (2.1)	20 (4.7)	0.59 (0.23–1.5)
≥ 13	129 (44)	222 (52)	1	Weekly milk consumption			
Age at menarche (years)				No	5 (1.7)	10 (2.4)	1
9–12.5	97 (34)	151 (36)	1.05 (0.72–1.5)	Yes	286 (98)	416 (98)	1.5 (0.50–4.4)
13	91 (32)	143 (30)	1	Diet without meat or fish			
13.5–19	94 (33)	127 (34)	1.2 (0.77–1.7)	No	277 (95)	419 (98)	1
History of irregular periods				Yes	14 (4.8)	8 (1.9)	2.8 (1.2–6.7)
No	205 (70)	289 (68)	1	Daily cigarettes, months 1–3			
Yes	87 (30)	138 (32)	0.89 (0.77–1.7)	None	249 (86)	361 (85)	1
Menstrual cycle length (days)				1–10	33 (11)	56 (13)	0.80 (0.50–1.3)
≤ 27	69 (25)	86 (21)	1.2 (0.82–1.7)	> 10	9 (3.1)	10 (2.3)	1.5 (0.59–3.9)
28–32	165 (61)	256 (64)	1	Passive maternal exposure to tobacco smoke during index pregnancy			
≥ 32	14 (5.2)	21 (5.2)	0.90 (0.42–1.9)	No	181 (62)	317 (74)	1
Irregular	24 (8.8)	39 (9.7)	0.95 (0.54–1.7)	Yes	110 (38)	110 (26)	1.8 (1.2–2.6)
Missing^a^	20	25		Characteristics of the child at birth			
History of spontaneous abortion				Birth order			
No	225 (77)	315 (75)	1	1	109 (40)	138 (44)	1
1	49 (17)	76 (18)	0.96 (0.64–1.4)	2	107 (40)	164 (20)	0.94 (0.65–1.3)
2	14 (4.8)	18 (4.3)	1.2 (0.60–2.5)	≥ 3	54 (20)	75 (37)	0.86 (0.55–1.3)
≥ 3	3 (1.0)	13 (3.1)	0.30 (0.08–1.08)	Missing^a^	22	50	
History of stillbirth				Weight for gestational age (percentile)			
No	289 (99)	417 (99)	1	< 2.5	12 (4.1)	6 (1.4)	3.1 (1.09–15)
Yes	2 (0.7)	4 (1.0)	0.94 (0.17–5.2)	2.5–9.9	35 (12)	15 (3.5)	3.1 (1.6–5.3)
Contraceptive use at conception				10.0–24.9	45 (15)	65 (15)	1.1 (0.67–1.9)
None	282 (97)	398 (94)	1	25.0–49.9	65 (22)	116 (27)	0.85 (0.55–1.4)
Combined oral contraceptives^b^	3 (1.0)	3 (0.7)	1.6 (0.33–8.3)	50.0–74.9	72 (25)	108 (25)	1
Progestin-only oral contraceptives	2 (0.7)	2 (0.5)	1.9 (0.25–14)	75.0–89.9	34 (12)	72 (17)	0.77 (0.45–1.3)
Nonhormonal method^c^	3 (1.0)	19 (4.5)	0.24 (0.07–0.81)	90.0–97.4	21 (7.2)	31 (7.3)	1.05 (0.55–2.0)
Time to index pregnancy (months)				≥ 97.5	7 (2.4)	12 (2.8)	0.86 (0.32–2.4)
< 6	187 (72)	244 (71)	1	Gestational age at birth (weeks)			
6–12	29 (11)	51 (15)	0.71 (0.41–1.2)	< 37	32 (11)	8 (1.9)	6.6 (2.9–15)
> 12	45 (17)	51 (15)	1.1 (0.71–1.7)	37–39	92 (32)	140 (33)	1.01 (0.72–1.4)
Missing^a^	31	81		40–41	133 (46)	211 (50)	1
Characteristics of the index pregnancy				42–45	34 (12)	66 (16)	0.80 (0.50–1.3)
High blood pressure				Neonatal jaundice			
No	236 (81)	392 (92)	1	No	190 (66)	320 (75)	1
Yes	55 (19)	35 (8.2)	2.8 (1.0–4.5)	Yes	100 (34)	107 (25)	1.5 (1.07–2.2)

a We give the number of missing values for variables with information missing for more than 10 subjects.

b Estrogen plus progestin.

c Nonhormonal IUD, condom, or rhythm method.

d First 3 months of pregnancy.

e Last 3 months of pregnancy.

**Table 2:** Associations between hypospadias and maternal, pregnancy, and birth characteristics: adjusted ORs from multivariable modeling in a matched case–control study, Denmark and Sweden, 2000–2005.^a^

Variable	OR (95% CI)	*p*-Value for trend	Variable	OR (95% CI)	*p*-Value for trend
Maternal age (years)			Passive maternal exposure to tobacco smoke during index pregnancy		
18–24	1.1 (0.51–2.5)		No	1	
25–29	1.3 (0.81–2.0)		Yes	1.9 (1.2–3.0)	
30–34	1		Weekly meat consumption		
≥ 35	0.62 (0.37–1.01)	0.06	No	2.4 (1.1–4.9)	
Maternal BMI, preindex pregnancy			Yes	1	
≤ 19	0.96 (0.55–1.6)		Weekly fish consumption		
20–24	1		None	1.4 (0.84–2.2)	
25–29	1.7 (1.02–3.0)		Less than once	2.7 (1.3–5.5)	
≥ 30	2.6 (1.2–5.7)	0.01	Once or twice	1	
Maternal education (years)			More than twice	0.88 (0.31–2.5)	0.02
≤ 9	1.4 (0.78–2.6)		Maternal diet without meat or fish, index pregnancy		
10–12	1.05 (0.66–1.7)		No	1	
≥ 13	1	0.56	Yes	4.6 (1.56–13)	
Contraceptive use at conception			Weight for gestational age (percentile)		
None	1		< 2.5	4.4 (1.2–16)	
Combined oral contraceptives^b^	1.3 (0.20–8.1)		2.5–9.9	4.6 (2.0–10)	
Progestin-only oral contraceptives	1.3 (0.13–13)		10.0–24.9	1.1 (0.61–2.1)	
Nonhormonal method^c^	0.22 (0.04–1.3)		25.0–49.9	0.86 (0.51–1.5)	
High maternal blood pressure, index pregnancy			50.0–74.9	1	
No	1		75.0–89.9	0.70 (0.36–1.4)	
Yes	2.0 (1.1–3.7)		90.0–97.4	1.09 (0.50–2.4)	
Proteinuria			≥ 97.5	0.66 (0.19–2.3)	
No	1		Gestational age at birth (weeks)		
Yes	1.6 (0.85–3.2)		< 37	9.1 (3.3–25)	
Maternal nausea, index pregnancy			37–39	0.80 (0.53–1.2)	
No	1.8 (1.2–2.8)		40–41	1	
Early pregnancy only^d^	1		42–45	0.82 (0.46–1.5)	
Late pregnancy only^e^	7.6 (1.09–53)		Neonatal jaundice		
Early and late pregnancy	1.2 (0.54–2.8)		No	1	
			Yes	1.2 (0.79–1.9)	

a We adjusted each OR for the other variables listed, except for contraceptive use at conception and proteinuria, which we added separately to the model. We introduced diet without meat or fish as an alternative to the variables weekly meat/weekly fish consumption.

b Estrogen plus progestin.

c Nonhormonal IUD, condom, or rhythm method.

d First 3 months of pregnancy.

e Last 3 months of pregnancy.
